# Simultaneous hypnotic intervention in preschool-aged monozygotic twin girls with persistent thumb sucking: a case report

**DOI:** 10.3389/fpsyg.2026.1889786

**Published:** 2026-09-17

**Authors:** Maks Tušak, Joseph Meyerson, Matej Tušak, Jasmina Markovič-Božič, Andrej Lapoša, Aleš Porčnik, Ivan Kneževič

**Affiliations:** 1 Department of Plastic, Reconstructive, Aesthetic Surgery and Burns, University Medical Centre Ljubljana, Ljubljana, Slovenia; 2 HypnoClinic, State-Approved Institute for Hypnosis Research, Education and Training, Tel Aviv, Israel; 3 Science and Research Centre Koper, Koper, Slovenia; 4 Clinical Department of Anaesthesiology and Surgical Intensive Care, University Medical Centre Ljubljana, Ljubljana, Slovenia; 5 Department of Anaesthesiology and Reanimation, Faculty of Medicine, University of Ljubljana, Ljubljana, Slovenia; 6 Department of Surgery, Faculty of Medicine, University of Ljubljana, Ljubljana, Slovenia; 7 Transplantation Centre, University Medical Centre Ljubljana, Ljubljana, Slovenia

**Keywords:** clinical hypnosis, habit cessation, relational, thumb sucking, twins

## Abstract

Persistent thumb sucking beyond early childhood may be associated with dental, dermatologic, and psychosocial complications and can prove resistant to conventional behavioral interventions. This case report describes the use of clinical hypnosis for persistent thumb sucking in nearly 6-year-old monozygotic twin girls. During the first session, Twin A and Twin B participated simultaneously in a developmentally adapted hypnotic intervention. At the second session, Twin A declined further hypnosis and engaged instead in a supportive therapeutic conversation, while Twin B received an additional hypnotic intervention. Following the completion of the intervention, the mother reported an almost complete cessation of thumb sucking in both children, with the improvement largely maintained at the 1-year follow-up. Beyond symptom reduction, the mother also reported increased differentiation between the twins and more individualized relational patterns within the family. Although outcomes were based on maternal report, this case highlights the potential clinical value of brief pediatric hypnosis and the possible relevance of twin and sibling dynamics in the treatment of shared habitual behaviors.

## Introduction

Clinical hypnosis has been widely recognized as an effective and developmentally congruent intervention in pediatric populations (Kohen and Olness, 2022). Children’s heightened imaginative capacity, natural dissociative abilities, and responsiveness to metaphor and imagery make hypnosis particularly suitable for addressing a range of emotional, psychosomatic, and behavioral difficulties. Hypnotic interventions have demonstrated enduring benefits across diverse pediatric conditions, including attentional disorders, chronic pain, enuresis, migraine headaches, functional gastrointestinal disorders, asthma, habit cough, anxiety-related somatic symptoms, and behavioral regulation difficulties—often in cases where conventional treatments have failed (Anbar, 2001; Anbar, 2002; Bayne, 2024; Kohen and Olness, 2022; Hudson, 2009; Gardner, 1981; Hammond, 1990; Sugarman, 1996; Lokshin et al., 1991; Richardson et al., 2006; Rienhoff et al., 2022). Although clinical reports and controlled studies have described beneficial outcomes of pediatric hypnosis across a range of conditions, the evidence base remains heterogeneous, with many studies involving relatively small samples, case series, or multicomponent interventions. Thus, while the available findings are encouraging, further systematic research is needed to clarify the strength of treatment effects and the specific mechanisms through which hypnosis may contribute to therapeutic change.

Within this broader clinical landscape, hypnosis has been applied successfully to pediatric habit and behavior disorders. Case series addressing conditions such as trichotillomania, nail biting and thumb sucking report high rates of symptom resolution and low relapse when hypnotherapy and self-hypnosis techniques are employed (Iglesias, 2003; Thomson, 2002; Hudson, 2009; Kohen and Olness, 2022).

These reports suggest that hypnosis may be useful in modifying habitual behaviors that serve regulatory or self-soothing functions; however, the limited number and methodological quality of available studies preclude firm conclusions regarding efficacy.

Thumb sucking represents one of the most common early self-soothing behaviors in infancy and early childhood, typically resolving spontaneously by approximately four to 5 years of age (Larsson, 1994). When the habit persists beyond the preschool years, however, it has been associated with dental malocclusions requiring orthodontic correction, speech difficulties, dermatologic irritation, and significant psychosocial consequences, including social stigma, peer ridicule, and exclusion (Warren and Bishara, 2002; Larsson, 1986; Fukuta et al., 1996). Conventional behavioral interventions—such as positive reinforcement strategies, reward charts, bitter-tasting deterrents, and intraoral appliances—are widely used. While often effective, these approaches frequently require sustained effort, may provoke resistance, and can cause discomfort or distress, particularly in younger children (Adair, 2003; Krishnappa et al., 2016). More recently, non-hypnotic interventions continue to show mixed outcomes. A recent randomized pilot study (Eltager et al., 2025) compared electronic habit-reminder wristwatches with fixed palatal cribs over a nine-month period. Although cessation rates were higher in the crib group (54.5%) than in the electronic reminder group (27.3%), the difference was not statistically significant, underscoring the limitations of reminder-based or aversive approaches and the need for alternative strategies.

Compared to behavioral and mechanical interventions, clinical hypnosis has been described as a gentle, child-centered approach that emphasizes collaboration, imagination, and developmental sensitivity. Contemporary hypnotic interventions for thumb sucking commonly integrate several complementary elements: the use of empowering and ego-strengthening suggestions that enhance the child’s sense of agency and self-regulation; the facilitation of awareness of automatic or habitual behaviors in order to enable their interruption; the incorporation of imaginative narratives and metaphoric storytelling tailored to the child’s developmental level; identification with a central character that allows for indirect learning and self-recognition; and the development of alternative soothing or calming behaviors to replace the habitual action. Together, these components aim to promote behavioral change while minimizing resistance and preserving the child’s subjective experience of competence and autonomy (Watkins and Barabasz, 2012; Hudson, 2009; Kohen and Olness, 2022).

Within this broader conceptual framework, only a small number of specific clinical cases have been reported. Erickson described a brief case of a child with persistent thumb sucking, in which he used indirect and paradoxical suggestions that utilized the symptom itself rather than confronting it directly, thereby facilitating behavioral change (Rosen, 2010). Tilton (1980) presented a case in which hypnotherapy was used concurrently to treat thumb sucking, enuresis, and encopresis in a single pediatric patient, demonstrating the flexibility of hypnosis in managing multiple co-occurring behavioral difficulties. More recently, Grayson (2012) reported a case of a 7-year-old girl whose persistent thumb sucking habit ceased following a single hypnotic session that combined habit validation, reframing of the behavior as developmentally outdated, role-model imagery, and suggestions promoting alternative comforting behaviors and emerging maturity.

Despite these contributions, the literature on hypnosis specifically for thumb sucking cessation remains limited, and its application within sibling or twin contexts is notably absent. This gap is particularly salient given that sibling dynamics—such as modeling, shared routines, mild competition, and mutual identification—may significantly influence both the maintenance and modification of habitual behaviors. In addition, the presence of a triangular interaction involving a significant adult (e.g., a parent) may further shape these dynamics and influence the child’s engagement with the therapist. Such relational configurations are likely to be especially pronounced in monozygotic twins, whose developmental synchrony and mutual attunement may amplify both interpersonal and therapeutic processes.

To our knowledge, no published report has described the simultaneous hypnotic treatment of monozygotic twin siblings for persistent thumb sucking. The present case report addresses this gap by examining hypnotic intervention with a pair of nearly 6-year-old identical twin girls, initially treated together and subsequently through a differentiated therapeutic process (see Table 1).

**Table 1:** Clinical course, intervention, and follow-up of persistent thumb sucking in monozygotic twins.

Time point	Twin A	Twin B
Baseline	Persistent thumb sucking across several daily situations and during sleep	Persistent thumb sucking across several daily situations and during sleep
Previous interventions	Encouragement, reminders, positive reinforcement, thumb guard; no sustained improvement	Same
Session 1	Joint hypnotic intervention	Joint hypnotic intervention
Home practice	Listened to Session 1 recording	Listened to Session 1 recording
Two-week follow-up	Greater maternally reported improvement; brief individual hypnotic attempt discontinued after 1–2 min; declined further hypnosis; supportive conversation	Improvement but residual thumb sucking; hypnotic intervention
Subsequent course	Continued improvement	Continued improvement
≈1-year follow-up	Minimal residual bedtime behavior reported	Minimal residual bedtime behavior reported

## Case description

The referral involved two healthy, nearly 6-year-old monozygotic twin girls who had been sucking their thumbs since infancy. For clarity and preservation of anonymity, the children are referred to throughout the report as Twin A and Twin B. Their developmental history was otherwise unremarkable. As their mother described it, the habit had gradually become woven into the fabric of their daily lives. According to their mother, the behavior occurred multiple times throughout the day and was consistently present before and during sleep, during stressful situations, and while riding in the car. This pattern was observed both at home and in kindergarten, where both girls consistently sucked their thumbs before and during daytime sleep. Thumb sucking also occurred during quiet, unstructured activities, such as watching television. Although the exact daily frequency was not recorded and no standardized measure of severity was used, its consistent occurrence in these situations, presence across multiple settings, and persistence despite repeated attempts to stop indicated a well-established habit. Over time, the family tried encouragement, positive reinforcement, gentle verbal reminders, and specially designed thumb-sucking deterrent devices (thumb guards), but none resulted in sustained improvement. The precise duration of each attempt was not documented. A dental examination revealed no consequences attributable to thumb sucking. No formal psychological assessment was performed.

The decision to seek professional help was driven largely by the mother’s growing concern. She was well aware of the potential long-term consequences of persistent thumb sucking, including malocclusion, alterations in palatal development, and improper tooth alignment. Beyond these dental considerations, she was also motivated by a broader developmental perspective, hoping to support her daughters in relinquishing the habit before entering formal schooling through an approach that would be both effective and non-invasive.

Because both twins presented with the same longstanding habit, shared many daily routines, and attended the consultation together, the therapist elected to begin with a joint intervention. This format was considered developmentally acceptable and allowed both children to engage with the same imaginative material while remaining together. Possible effects of sibling modeling or mutual influence were clinical considerations rather than formally assessed treatment mechanisms.”

The literature on twin development suggests that such parallel interventions may capitalize on mutual identification and modeling processes, which are especially pronounced in monozygotic twins (e.g., Kohen and Olness, 2022; Segal, 2012).

The first session was conducted with both children present and used a playful, imaginative format adapted to their developmental level. Hypnosis was introduced in simple terms as ‘relaxed imagining’ or daydreaming. The girls were invited to close their eyes, if comfortable, and imagine the inside of their eyelids as a screen on which they could watch a favorite cartoon. Both children appeared engaged and increasingly absorbed in the exercise, with observable relaxation of facial musculature.

During the hypnotic work, suggestions were individualized yet coordinated, aligning with the girls’ shared developmental stage and personal interests. They were invited to imagine themselves preparing to go to school and becoming “big girls,” with the idea that big girls no longer need to suck their thumbs. This framing intended to emphasized growth and agency rather than prohibition, reinforcing a positive and empowering self-image.

Crucially, the suggestions were accompanied by explicit reassurance. The girls were told that they were not expected to stop sucking their thumbs immediately—not the next day, nor even the following week. Instead, they were encouraged to allow the change to occur when they themselves felt ready. This approach reduced performance pressure and supported intrinsic motivation—a principle originally articulated by Milton H. Erickson and described in detail by Haley in Uncommon Therapy through his accounts of Erickson’s clinical work, and later emphasized in the pediatric hypnosis literature (Haley, 1973; Kohen and Olness, 2022; Watkins and Barabasz, 2012).

In a further playful intervention, the therapist invited the girls to imagine their ballet teacher sucking her thumb and asked, with gentle humor, whether that would not be funny (Erickson et al., 1976). The children responded with laughter, and the image helped render the habit incongruent with admired, competent figures, subtly strengthening their motivation to relinquish it.

An audio recording of the first intervention was provided for optional home practice. According to the mother, both twins listened to the recording twice during the two-week interval before the second meeting, particularly before sleep. The second meeting was also recorded. Following that meeting, Twin B listened to the recording once more approximately 1 week later. According to their mother, the girls engaged attentively with the audio recordings.

Two weeks later, a second meeting was conducted. According to the mother, both children had reduced their thumb sucking, with greater improvement reported for Twin A.

At the beginning of the second session, twin A initially engaged with the suggestions for approximately one to 2 min but then became increasingly active, walked around the room, spoke aloud, and ceased attending to the exercise. The hypnotic intervention was therefore discontinued without further attempts to re-engage her. She clearly stated that she did not wish to participate in further hypnosis. Her preference was respected, and she instead engaged in supportive therapeutic conversation with the therapist. Twin B was then invited to participate individually in another imaginative exercise and remained engaged throughout the session.

During the supportive conversation with Twin A, discussion of her sister’s individual therapeutic attention elicited a mild reaction that the therapist understood as possible jealousy or concern about exclusion. The therapist acknowledged her reaction and reassured her that her progress did not require an additional hypnotic intervention. The interpretation of this interaction as reflecting differentiation or a triangular relational process represents a clinical hypothesis rather than a formally assessed phenomenon.

Rather than challenging or interpreting these feelings directly, the therapist acknowledged and contained them, reassuring the child that her progress was evident and that she did not need additional session of hypnosis in order to succeed. The session concluded with validation of her autonomous choice and reinforcement of her independent developmental trajectory. This handling of both the need for differentiation and the triangular transference appeared to preserve the therapeutic alliance, support self-confidence, and consolidate behavioral change, while allowing each twin to proceed along her own therapeutic path within a shared relational field.

## Results

The children’s mother reported that on the evening following the first session, both girls fell asleep without engaging in thumb sucking—an outcome that had not been observed previously. It should be noted that the parents reported no substantial change in their behavioral approach or reinforcement strategies following the intervention. By the time of the second session, the frequency and intensity of the behavior had already decreased in both children.

By the time of the second session, the frequency and intensity of thumb sucking had decreased in both children. The improvement was more pronounced in Twin A, who subsequently declined further hypnosis, whereas Twin B continued to suck her thumb intermittently, particularly during periods of stress or fatigue. Over the subsequent weeks, thumb sucking continued to diminish in both girls, approaching near extinction. At follow-up approximately 1 year later, the mother confirmed that the improvement had been maintained. On rare occasions, one or both children would briefly place a thumb in their mouth while falling asleep; however, thumb sucking no longer occurred during the night. Prior to treatment, the behavior had been persistent throughout the night, even after active attempts at removal.

In addition to the reduction in thumb sucking, the mother noted a secondary change in the twins’ relational patterns. Following the intervention, the girls appeared more differentiated from one another, expressing clearer individual preferences and needs. This increased differentiation was also reflected in more distinct and individualized interactions with their mother, who reported relating to each child in a less symmetrical and more attuned manner. While not an explicit therapeutic target, this shift toward greater individuation was experienced by the family as a positive and meaningful outcome.

At follow-up, the children’s mother expressed high satisfaction with the overall outcome, reporting a near-complete and sustained cessation of the thumb sucking behavior alongside these relational changes. In light of the stability of the gains and the clinical success of the intervention, no additional hypnosis sessions were deemed necessary.

## Discussion

The present case describes marked and largely sustained maternally reported improvement in persistent thumb sucking following a brief, multicomponent intervention involving an initial joint hypnotic session, developmentally adapted imagery and suggestion, home audio exposure, and a subsequent differentiated therapeutic encounter. Given the uncontrolled case-report design and absence of standardized outcome measures, the findings should be regarded as preliminary and hypothesis-generating rather than evidence of efficacy.

These findings are broadly consistent with existing literature suggesting that hypnosis may offer a non-invasive and developmentally sensitive approach to pediatric habit disorders (Watkins and Barabasz, 2012; Kohen and Olness, 2022). Beyond the observed behavioral improvement, the present case adds a distinctive clinical perspective by illustrating the use of hypnosis within a sibling and twin context—an area that remains scarcely represented in the literature—and raises clinically relevant questions regarding the possible role of shared relational processes in treatment.

A central and innovative aspect of this report is the use of a simultaneous hypnotic intervention with identical twins. While individual hypnotic treatment for thumb sucking has been described in the literature, joint hypnotic work with siblings—and particularly with monozygotic twins—has rarely been described. A distinctive feature of the case was the initial simultaneous intervention with monozygotic twins. Joint treatment created a shared therapeutic context in which modeling, comparison, and mutual influence could potentially occur. These processes were not independently assessed, however, and their contribution to behavioral change remains hypothetical Developmental and psychoanalytic literature on twins suggests that these relational processes are especially salient among identical twins and may significantly influence symptom maintenance and change (Segal, 2012; Lewin, 2018). The present case suggests that such dynamics can be actively and constructively engaged within the hypnotic setting.

The few available comparative reports further underscore the novelty of this approach. Watson et al. (2002) demonstrated that modifying thumb sucking behavior in one sibling could lead to parallel changes in an untreated sibling, highlighting the systemic nature of habitual behaviors within families. Túry et al. (2011) reported successful tandem hypnosis in adult monozygotic twins with bulimia nervosa, suggesting that shared hypnotic work may be particularly effective when relational and dissociative processes are central. Although these studies differ substantially in population and clinical focus, they converge with the present case in indicating that interventions addressing symptoms within a relational framework may yield amplified therapeutic effects.

Another important contribution of this case lies in its demonstration of flexibility and individualization within a shared therapeutic frame. When one twin showed marked improvement and declined participation in a second hypnotic session, this preference was respected and addressed through supportive conversation rather than overridden. This stance preserved the child’s sense of agency and allowed for differentiation within the twin relationship—a developmental task of particular importance for identical twins (Segal, 2012; Lewin, 2018). The different responses of the twins during the second meeting also illustrate the importance of preserving individual choice within an initially shared treatment format. Twin A’s decision not to continue hypnosis was respected, while Twin B’s brief individual attempt was discontinued when she disengaged. The mother subsequently perceived greater differentiation between the girls. Because individuation was neither a predefined treatment target nor formally measured, no conclusion can be drawn regarding whether the intervention contributed to this perceived change.

The mild jealousy or sense of exclusion observed during the second meeting may be understood clinically within a relational or triangular framework. However, this interpretation was generated by the therapist and was not independently assessed. It is therefore best regarded as one possible formulation of the interaction rather than as evidence of a specific mechanism of change.”

The intervention also illustrates the use of developmentally attuned, symbolic, and playful strategies. Indirect imagery and humor allowed the children to reconsider the habit without confrontation or pressure, fostering engagement and intrinsic motivation. Such techniques are well supported in the pediatric hypnosis literature and may be particularly valuable when working with young children and shared symptoms within a family context (Kohen and Olness, 2022; Watkins and Barabasz, 2012).

It should be acknowledged that alternative explanations may also have contributed to the observed improvement. The act of seeking professional help, the symbolic meaning of entering therapy, and the children’s developmental proximity to school entry may have reinforced their motivation toward increased maturity independent of hypnotic mechanisms per se. The parents did not report substantial changes in their behavioral management strategies following the first session. Nevertheless, expectancy effects, therapeutic ritual, and systemic family dynamics cannot be fully ruled out in a single-case report.

Notably, the report includes a long-term follow-up nearly 1 year after treatment, demonstrating sustained improvement with only minimal and transient residual behavior. The durability of change strengthens the clinical significance of the intervention and s consistent with the possibility that brief, relationally informed hypnotic work can lead to stable behavioral outcomes.

In sum, this case highlights the potential advantages of incorporating sibling and twin dynamics directly into hypnotic treatment planning. It suggests that, particularly in identical twins or siblings presenting with similar habitual behaviors, joint or coordinated hypnotic interventions may offer a unique and efficient therapeutic pathway. By integrating individual responsiveness with shared relational processes, this approach expands the clinical possibilities of pediatric hypnosis and invites further systematic investigation into its applicability across family-based contexts.

### Limitations

Several limitations substantially restrict interpretation of this case. First, outcome assessment relied on maternal report; no standardized baseline or follow-up measure, behavioral diary, independent observation, or blinded assessment was used. Parental expectations, attention to the behavior, recall, or interpretation may therefore have influenced the reported magnitude of change. Second, the girls were approaching 6 years of age, an age at which spontaneous developmental reduction of thumb sucking may occur. Third, seeking professional treatment may itself have altered parental attention and family responses. Nonspecific therapeutic factors, including novelty, therapist attention, therapeutic alliance, expectancy, and increased motivation, may also have contributed. Fourth, exposure to the recorded intervention may have reinforced change independently of the in-session hypnotic intervention.

In addition, the intervention was inherently multicomponent. Hypnotic imagery and suggestion were combined with developmental reframing, humor, therapist attention, sibling presence, supportive conversation, and home audio exposure. The present report therefore cannot determine which component, or combination of components, accounted for the observed change, nor can it establish a specific causal role for hypnosis.

## Conclusion

This case suggests that simultaneous and individualized hypnosis may represent a promising approach for persistent thumb sucking in monozygotic twins. Meaningful and sustained behavioral change was achieved through a minimal number of sessions, supplemented by guided audio recordings for home use. Beyond symptom reduction, the intervention appeared to engage relational processes inherent in the twin relationship, including mutual influence and emerging differentiation, which may have contributed to therapeutic responsiveness. These findings underscore the potential value of developmentally attuned hypnotic interventions for young children and highlight the clinical relevance of considering sibling—particularly twin—dynamics when treating shared habitual behaviors. Further controlled research is required to determine efficacy and to clarify the role of sibling and twin dynamics in treatment outcomes.”

## Data Availability

The raw data supporting the conclusions of this article will be made available by the authors, without undue reservation.
